# Characterization, treatment and prognosis of retinoblastoma with central nervous system metastasis

**DOI:** 10.1186/s12886-018-0772-8

**Published:** 2018-04-23

**Authors:** Huimin Hu, Weiling Zhang, Yizhuo Wang, Dongsheng Huang, Jitong Shi, Bin Li, Yi Zhang, Yan Zhou

**Affiliations:** 1 Department of Pediatrics, Beijing Tongren Hospital, West South road 2, Yizhuang Economic and Technological Development Zone, Daxing District, Beijing, 100176 China; 2 Department of Ophthalmology, Beijing Tongren Hospital, Capital Medical University, Beijing, 100176 China

**Keywords:** Retinoblastoma, Central nervous system metastasis, Pediatric

## Abstract

**Background:**

Retinoblastoma is the most common primary intraocular tumor and more and more attention has been paid to the developing countries. This study was aimed to evaluate the clinical features, treatment, and prognosis of retinoblastoma patients with central nervous system (CNS) metastasis in Beijing Tongren Hospital, one of the largest tertiary eye centers in China.

**Methods:**

Clinical data of 31 consecutive retinoblastoma patients with CNS metastases, who were diagnosed at the Department of Pediatrics in Beijing Tongren Hospital between September 2005 and December 2015, were retrospective analyzed.

**Results:**

The median age at presentation was 29 months (range from 5 to 108 months). Magnetic resonance imaging (MRI) results indicated that 16 patients (56.6%, 16/31) presented with meningeal involvement, 12 (38.7%, 12/31) presented with intracranial mass, 11 (35.5%, 11/31) presented with thickened optic nerve, and 5 (16.1%, 5/31) presented with concurrent meningeal and spinal cord membrane involvement. Retinoblastoma cells were detected in the cerebrospinal fluid (CSF) of 12 patients (44.4%, 12/27). Laboratory examinations on the blood and CSF were performed for 11 patients who had received six cycles of systemic chemotherapy, indicated that the serum level of neurone-specific enolase (NSE) after chemotherapy was significantly lower than that before chemotherapy (*P <* 0.05). At the end of the follow-up, 25 patients were dead with a median survival time of 6 months (1 d – 21 months), and 6 cases were alive and continued to receive treatment.

**Conclusion:**

Our results were basically consistent with previous reports in the developing countries, and it could be guidance for clinical treatment, prognosis and prevention of CNS metastases in retinoblastoma.

**Electronic supplementary material:**

The online version of this article (10.1186/s12886-018-0772-8) contains supplementary material, which is available to authorized users.

## Background

Retinoblastoma is the most common intraocular malignancy in infancy and childhood, with an reported incidence of 1 per 15,000–20,000 live births [1], whereas accounts for 3% of all pediatric cancers [2]. The overall survival rate of retinoblastoma was reported to exceed 95% when children were early diagnosed with localized intraocular phase [3, 4]; however, delayed diagnosis and treatment, which is common situation in the developing countries, may lead to extraocular metastasis, visual loss and death. Meanwhile, secondary to the advanced retinoblastoma cases with central nervous system (CNS) metastasis is associated with exceedingly poor prognosis [5, 6]. Therefore, treatment of retinoblastoma patients with CNS metastases should be tailored to the individual according to the clinical conditions and nature of the tumors. Conventional therapies generally include systematic chemotherapy, enucleation, radiotherapy, even autologous peripheral blood stem cell transplantation (APBSCT), etc. [5].

Management of metastatic retinoblastoma is gaining more and more attention in China, which is a developing country with the largest population. The objective of current study was to evaluate and summarize the clinical features, treatment, and prognosis of retinoblastoma patients with CNS metastases, who were treated at the Department of Pediatrics in Beijing Tongren Hospital, one of the largest and best-known tertiary eye centers in China.

## Methods

A total of 1404 retinoblastoma patients were received at the Department of Pediatrics in Beijing Tongren Hospital, Beijing, China, between September 2005 and December 2015. Then 31 consecutive retinoblastoma patients who diagnosed with CNS metastasis were eligible for this study and clinical data were analyzed retrospectively. The study protocol was approved by the Medical Ethics Committee of the Beijing Tongren Hospital, and all participants gave their written informed consent. Orbital computed tomography (CT) scan, cranial CT scan and/or cranial magnetic resonance imaging (MRI) examination were performed in all patients. Trilateral retinoblastoma was excluded from the study. American Joint Committee on Cancer TNM clinical classification system [7] was used for the assessment of the whole patient by extent of extraocular disease (Additional file 1: Table S1). Orbital enhanced MRI, cranial MRI, contrast enhanced CT scan of the lungs, bone marrow cytology, hepatobiliary lymph node ultrasonography, superficial lymph nodes ultrasound and cerebrospinal fluid cytology examination were performed for extraocular patients in order to definite specific TNM stages.

Multi-drug combination chemotherapy was adopted in our treatment, including vincristine (1.5 mg/m^2^, day 1), etoposide/teniposide (100 mg/m^2^, day 2–3), carboplatin (560 mg/m^2^, day 1), and cyclophosphamide (65 mg/kg, day 2). In each cycle of the chemotherapy, lumbar puncture and intrathecal injection were performed for every patient with methotrexate (MTX), cytosine arabinoside (Ara-c), and dexamethasone (Dex) according to the following regimen: age < 12 months: MTX (5.0 mg), Ara-c (12.0 mg), Dex (2.0 mg); age 12–24 months: MTX (7.5 mg), Ara-c (15.0 mg), Dex (2.0 mg); age 25–35 months: MTX (10.0 mg), Ara-c (25.0 mg), Dex (5.0 mg); age ≥ 36 months: MTX (12.5 mg), Ara-c (35.0 mg), Dex (5.0 mg). Routine biochemistry of Cerebrospinal fluid (CSF) and cytology examinations were performed; moreover, neuron specific enolase (NSE) in serum and CSF of patients were analyzed using the electrochemiluminescence with the NSE Kit (Roche, US).

Cranial and orbital contrast-enhanced MRI examinations were routinely performed every two chemotherapy cycles. Children patients with CNS metastases were received radiation therapy at the dose of 40 Gy after chemotherapy. Tumor spreads through the cerebrospinal fluid was generally required craniospinal irradiation, and the dose of radiotherapy is not more than 40 Gy. Adverse reactions to radiotherapy and chemotherapy were evaluated according to CTCAE standard [8]. APBSCT treatment (APBSCT regimen: CBP 425 mg/ (m^2^.d); from − 6 to − 3 days + VP-16: 338 mg/ (m^2^.d); From − 6 to − 3 days + CTX: 1.5 g / (m^2^.d) was performed for 2 patients after achieving a stable disease condition.

Statistical analysis was performed using SPSS software (version 20.0, IBM, USA). For continuous variables, data were presented as mean ± standard deviation or median (Q25, Q75), and were compared using Student’s paired t-tests or Wilcoxon matched-pairs signed-rank test. A value of *P* < 0.05 was considered statistically significant. Kaplan-Meier survival curve was plotted to analyze the survival time after the diagnosis of CNS metastasis, the median survival time was also calculated.

## Results

**Fig. 1:**
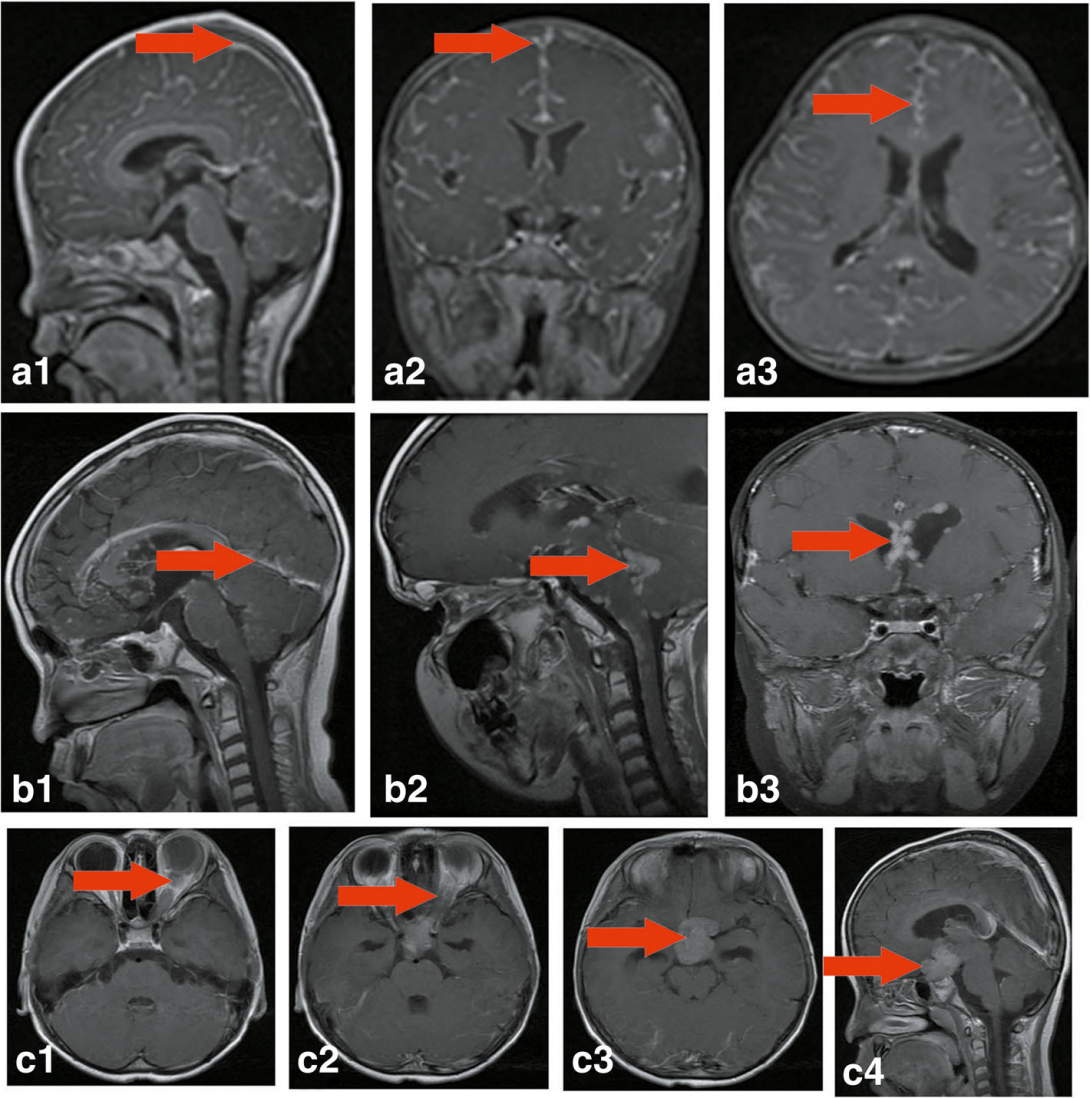
Representative MRI results of retinoblastoma patients with CNS metastasis. (**a**1–3) Head contrast-enhanced MRI: visible diffuse enhancement of the intracranial pial from sagittal, axial, and coronal imaging, respectively, cervical spinal cord membrane also show enhancement. (**b**1–3) Head and orbital contrast-enhanced MRI: sagittal image shows thickened meninges of the anterior, middle and posterior cranial fossa, brain stem surface, and the cerebellum (**b**1); and orbital MRI images show enhanced signaling of nodules, possibly spreading via the cerebrospinal fluid (**b**2–3). (**c**1–4) Head MRI: signs of optical nerve, optic chiasma, and supra sella cistern involvement, respectively (**c**1–3); visible mass formed in the supra sella cistern, and enhanced signal of the cerebral falx (**c**4). MRI: magnetic resonance imaging; CNS: central nervous system

**Fig. 2:**
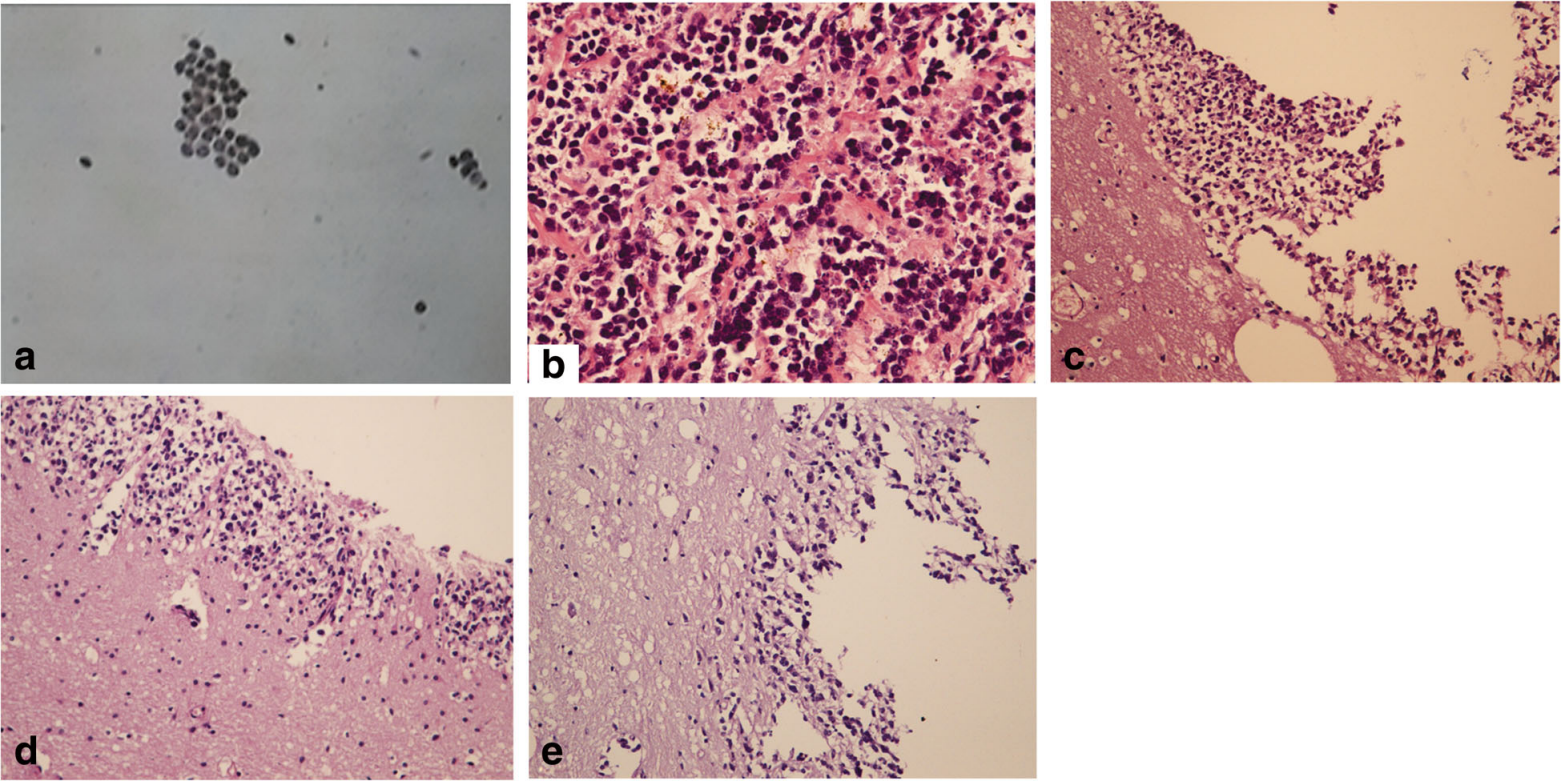
Representative images of CSF cytology and histopathology in retinoblastoma patients with CNS metastasis. Retinoblastoma cells as shown in the CSF smear (**a**, × 200), and tumor cells spreading in the optic nerve (**b**, × 200), left temporal lobe (**c**, × 100), mesencephalon (**d**,× 100), and cerebellomedullary (**e**,× 100) as stained by haematoxylin and eosin. CSF: cerebrospinal fluid; CNS: central nervous system

Of the enrolled 31subjects, 14 retinoblastoma cases were diagnosed CNS metastasis at the time of first RB diagnosis. 17 retinoblastoma cases were detected CNS metastases at some later point, the median time/range from RB diagnosis to CNS metastasis was 9 months (3-23 months). 23 (74.2%) were unilateral, and 8 (25.8%) were bilateral advanced retinoblsatoma. The median age at the time of patient visit was 29 months (range from 5 to 108 months). The median lag period of 31 patients between first symptom and initiation of treatment was 3 months. The median lag period of subjects at T stage (17/31) between first symptom and initiation of treatment was 1 month, whereas the median lag period was 6 months for patients at M stage (14/31). Detailed clinical characteristics are shown in Table 1. MRI results showed that the proportion of subjects with meningeal involvement accounts for 56.6% (16/31), followed by presented with, 12 intracranial mass (38.7%, 12/31) and thickened optic nerve (35.5%, 11/31).Representative MRI results of retinoblastoma patients with CNS metastases are also shown in Fig. 1. In addition, a positive rate of retinoblastoma cells in CSF cytology was 44.4% (12/27). Two patients’ parents were unable to accept eye enucleation, although all images of retinoblastoma showed an indication of enucleation. Images of CSF cytology and histopathology examinations are shown in Fig. 2. Autopsy was suggested and performed in one patient, who was the first retinoblastoma patient with orbital exente ation diagnosed in our department, but no metastasis was found by cranial MRI. Then the patient was dead after six months of chemotherapy. The autopsy results indicated that the optic nerve, left temporal lobe, antennal lobe, mesencephalon, and cerebellomedullary were all affected. Complications of chemo-radiotherapy were evaluated, and mainly including headache, vomit, fever, and myelo suppression, and all attenuated after the treatment. Neither hemorrhagic cystitis nor peripheral nervous system damage was found.

**Table 1:** Clinical characteristics of retinoblastoma patients with CNS metastasis

Characteristics	Data
Gender (male), n (%)	14 (45.2)
Age at presentation (months), median (range)	29 (5 to 108)
Laterality	
Bilateral, n (%)	8 (25.8)
Unilateral, n (%)	23 (74.2)
Right eyes, n (%)	14 (60.9)
Family history, n (%)	1 (3.2)
Lag period between first symptom and initiation of treatment (months), median (range)	3 (0.03–21)
T stages at first diagnosis	1 (1–24)
M stage at first diagnosis	6 (0.5–12)
TNM stages at first diagnosis, n (%)	
T1	3 (9.7)
T2	0
T3	10 (32.3), (T3a:6; T3b:4)
T4	4 (12.9), (T4a:3; T4b:1)
M	14 (45.2)
MRI features, n (%)	
Meningeal involvement	16 (56.6)
Meningeal and spinal cord membrane involvement	5 (16.1)
Intracranial mass	12 (38.7)
Thickened optic nerve	11 (35.5)
Involvement of other systematic organs, n (%)	
CNS with intraorbital involvement	12 (38.7)
CNS with bone	10 (32.3)
CNS with bone marrow	1 (3.2)
CNS with lung	2 (6.5)
CNS with pleura	1 (3.2)
CNS with mass of the lateral wall of pelvis	1 (3.2)
Surgery	
Enucleation	24 (77.4)
Exenteration	5 (16.1)
CSF cytology positive	12 (44.4, 12/27)

*CNS*: central nervous system; *MRI*: magnetic resonance imaging; *CSF*: cerebrospinal fluid

**Fig. 3:**
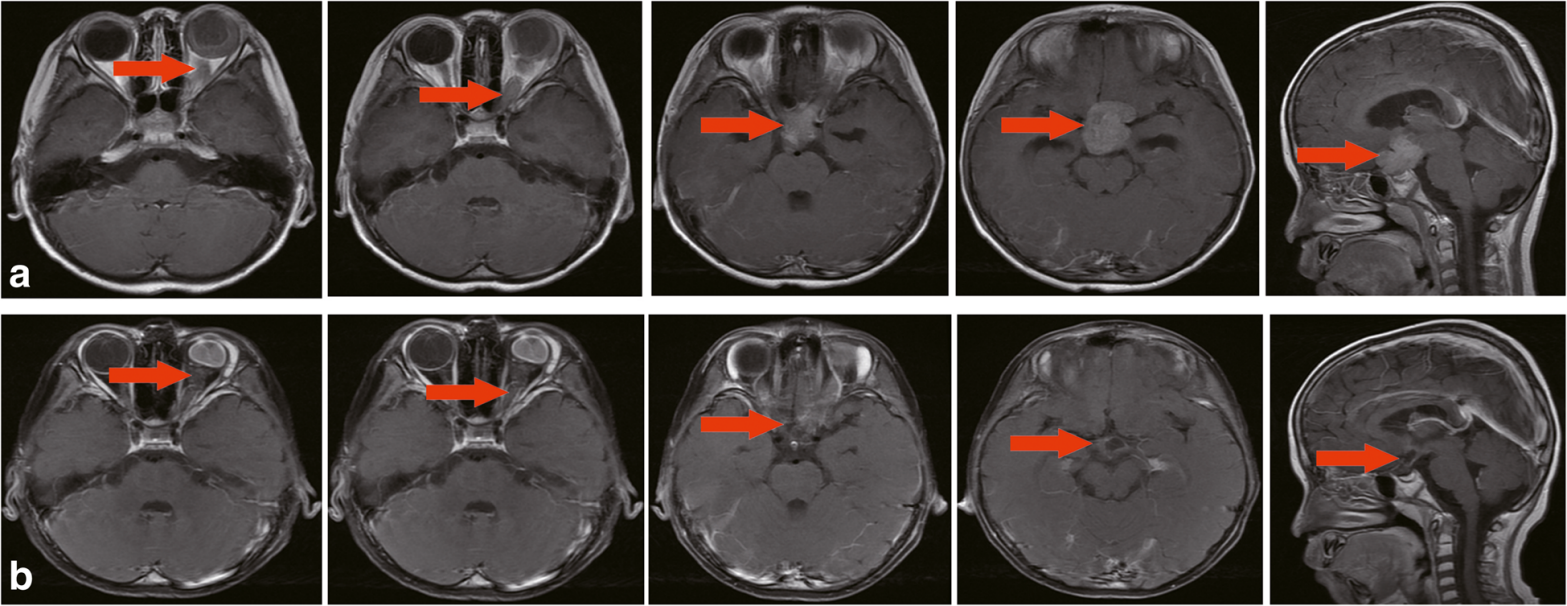
Contrast-enhanced MRI images of tumor regression and attenuated meningeal enhancement after chemotherapy. Coronal, and sagittal images show the mass in the supra sella cistern and enhanced signal of cerebral falx before chemotherapy (**a**), which were improved after chemotherapy (**b**). MRI: magnetic resonance imaging

Laboratory examinations were performed for the serum and CSF samples after six cycles of systemic chemotherapy (11 cases). As shown in Table 2, the level of neurone-specific enolase (NSE) in serum was found to be significantly decreased after chemotherapy (31.70 (26.50, 45.60) μg/L versus 91.20 (45.50, 6.20) μg/L, *P <* 0.05), and white blood cell count in CSF was also reduced after chemotherapy (4.27 ± 1.60 × 10^6^/L versus 80.36 ± 18.69 × 10^6^/L, *P <* 0.05). Contrast-enhanced MRI results showed tumor size and meningeal enhancement were attenuated after treatment (Fig. 3).

**Table 2:** Laboratory examination results of the blood and CSF samples from 11 patients after six cycles of systemic chemotherapy

Parameters	Normal range	Pre-chemotherapy	Post-chemotherapy
Serum NSE, μg/L	0–16.3	91.20 (45.50, 6.20)	31.70 (26.50, 45.60)*
CSF NSE, μg/L	0–16.3	54.60 (31.50, 196.40)	24.00 (9.60, 51.20)
CSF protein, g/L	0.15–0.45	0.34 (0.08, 0.83)	0.30 (0.07, 0.45)
CSF white blood cell, ×10^6^/L	0–10	80.36 ± 18.69	4.27 ± 1.60*

Data are presented as median (Q25, Q75), or mean ± standard deviation. *P < 0.05 versus pre-chemotherapy. *CNS*: central nervous system; *NSE*: neurone-specific enolase; *CSF*: cerebrospinal fluid

**Fig. 4:**
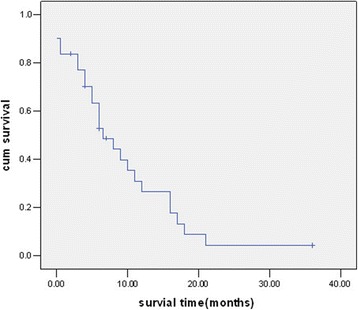
Kaplan-Meier survival curve of retinoblastoma patients with CNS metastasis. CNS: central nervous system

All patients were followed up until March 31, 2016, and at the end point death. Among the 31 cases, 25 patients were dead with a median survival time of 6 months (1 day – 21 months), and 6 patients with disease were alive with a median follow-up time of 6 months (2–36 months) and were continually under therapy. Kaplan-Meier survival curve is plotted as shown in Fig. 4.

## Discussion

In the present study, among the 31 cases of retinoblastoma patients with CNS metastasis, 12 cases were found to have intraorbital involvement; consistently, it was reported that retinoblastoma patients with intraorbital involvement tend to have systemic metastasis and a high mortality rate, and the CNS metastasis is the most common [6]. As previously reported, retinoblastoma with invasion of the postlaminar optic nerve is at high risk of metastasis and death [9]. We also found that the percentage of optic nerve involvement was as high as 35.5% (11/31). The median lag period between first symptom and initiation of treatment for patients with M stage is 6 months, whereas 1 month for T stage patients. There has been demonstrated that the longer lag period was associated with higher risk of CNS metastasis [10]. Thus, shortening the lag period has been considered as an important target of treatment.

It is well known that retinoblastoma is the most common intraocular malignancy in childhood in developed countries. Secondary to late presentation, advanced retinoblastoma with metastatic disease is more common in developing countries and is associated with delayed diagnosis and extremely poor prognosis. Secondary to the poor overall prognosis of retinoblastoma cases with CNS metastases can be classified metastatic retinoblastoma patients into those presenting with or without CNS disease. MRI can serve as a useful adjunct method for diagnosis in retinoblastoma with CNS metastasis [11]. Our results show that, meningeal and/or spinal cord membrane involvement, and intracranial mass were most common image features in retinoblastoma patients with CNS metastasis, which is consistent with a previous report [7]. The metastasis occurrence was mainly due to direct invasion of the optic chiasma, intracranial optical nerve, supra sella cistern, meninges, even locally brain parenchyma. Meningeal involvement combined with spinal cord membrane involvement might be due to the meninges invasion and/or the spreading of CSF. Meanwhile, other metastasis pathways such as hematogenous metastasis might be also involved cases with bone and bone marrow metastasis, and pulmonary and pleural metastasis. Thus systemic contrast-enhanced MRI is necessary for the diagnosing and monitoring.

CSF examination was regarded as a gold standard for diagnosing CNS metastasis in retinoblastoma patients, and repeated could increase the sensitivity [12, 13]. In the present study, a positive rate of CSF cytology (44.4%) was consistent with reported previously. One of the reasons for negative results (15 cases) in our study was not received repeated CSF examinations. Therefore, we suggest that repeated CSF cytology examination can elevate the detection rate of CNS metastasis during the process of treatment.

NSE, a 78-kDa dimeric γ-isoenzyme of the glycolytic enzyme enolase, is localized predominately in the cytoplasm of neurons and neuroendocrine cells. The elevated NSE levels have been shown in retinoblastoma patients. Thus, NSE levels of CSF and the serum are the standard at our institution. The CSF was taken from the patients with optic nerve invasion and CNS metastasis before intrathecal injection to perform CSF and biochemical routine examinations, also included NSE and pathological examinations [14, 15]. In the present study, NSE levels were found to be elevated in both serum and CSF samples of retinoblastoma patients with CNS metastasis. It might be more NSE was released into the serum and CSF due to tumor invasion. Meanwhile, retinoblastoma cells might also contribute to the NSE secretion. In addition, NSE levels were decreased after systematic chemotherapy, indicating that NSE might have a potential role in the diagnosis and therapeutic effect.

Regarding the fact that extraocular retinoblastoma is very common in the developing countries. Palliative-care protocols such as chemo- and radiotherapy are urgently needed, also it is necessary to optimize. Based on previous reports [16–19], chemotherapy provides a good palliation of extraocular retinoblastoma, even multi-drug combination chemotherapy was adopted in our department, including vincristine, etoposide/teniposide, carboplatin, and cyclophosphamide, combined with intrathecal injection treatment in each cycle of the chemotherapy. Our results also showed that NSE level in the serum and white blood cell count were significantly reduced after chemo and radiotherapy [20]. Furthermore, findings of the present study are consistent with Palma et al. who reported a significantly improved in treatment with APBSCT [21], suggesting it play a potential role in the treatment of retinoblastoma.

Although varied in different studies, the prognosis of retinoblastoma with CNS metastasis is fairly poor, especially in the developing countries [6, 22, 23]. Consistently, in our study, the median survival time was as short as 6 months; this might be due to missed diagnosis or mismanagement before the referral, and denial of management by parents, etc.

## Conclusion

In the present study, we reported the characteristics, treatment, and prognosis of retinoblastoma with CNS metastasis at the Department of Pediatrics in Beijing Tongren Hospital,.one of the largest and best-known tertiary eye centers in China. The outcomes of our study were similar to those expected in the developing countries. Since the early diagnosis and timely treatment are vital for a good prognosis, thus, much more efforts are needed to increase the public awareness, implement appropriated education programs for pediatricians and ophthalmologists, and improve the health care accessibility.

## Additional files

Additional file 1: **Table S1.** AJCC TNM Staging System. (DOCX 18 kb)

## Abbreviations

APBSCT: Autologous Peripheral Blood Stem Cell Transplantation
CNS: Central Nervous System
CSF: Cerebrospinal Fluid
CT: Computed Tomography
MRI: Magnetic Resonance Imaging
MTX: Methotrexate
NSE: Neuron Specific Enolase
NSE: Neurone-specific Enolase
