# Supplementary material for: Characterization, treatment and prognosis of retinoblastoma with central nervous system metastasis
Source: BMC Ophthalmol. 2018 Apr 23;18:107. doi: 10.1186/s12886-018-0772-8 (PMC5914066; doi:10.1186/s12886-018-0772-8)
Supplement: Supplementary file 1 — Table S1. AJCC TNM Staging System. (DOCX 18 kb) [file 12886_2018_772_MOESM1_ESM.docx]

Table S1.

| AJCC TNM Staging System | | |
| --- | --- | --- |
| T stages |  |  |
|  | pT1 | Tumor confined to the eye with no optic nerve or choroidal invasion |
|  | pT2 | Tumor with minimal optic nerve and/or choroidal invasion |
|  | pT2a | Tumor superficially invades optic nerve head but does not extend past lamina cribrosa, or tumor exhibits choroidal invasion |
|  | pT2b | Tumor superficially invades optic nerve head but does not extend past lamina cibrosa and tumor exhibits focal invasion |
|  | pT3 | Tumor with significant optic nerve and/or choroidal invasion |
|  | pT3a | Tumor invades optic nerve past lamina cribrosa but not to surgical resection line, or tumor exhibits massive choroidal invasion |
|  | pT3b | Tumor invades optic nerve past lamina cribrosa but not to surgical resection line and exhibits massive choroidal invasion |
|  | pT4 | Tumor invades optic nerve to surgical resection line or exhibits extraocular extension elsewhere |
|  | pT4a | Tumor invades optic nerve to resection line, but no extraocular  extension identified |
|  | pT4b | Tumor invades optic nerve to resection line, and extraocular extension identified |
| N stage | pN1 | Regional lymph node involvement (preauricular, cervical) |
| M stages |  |  |
|  | pM1 | Metastasis to sites other than central nervous System |
|  | pM1a | Single lesion |
|  | pM1b | Multiple lesions |
|  | pM1c | CNS metastasis |
|  | pM1d | Discrete masses without leptomeningeal and/or CSF involvement |
|  | pM1e | Leptomeningeal and/or CSF involvement |

AJCC, American Joint Committee on Cancer; CNS, central nervous system; CSF, cerebrospinal fluid
